# Residual fibroglandular breast tissue after mastectomy is associated with an increased risk of a local recurrence or a new primary breast cancer"

**DOI:** 10.1186/s12885-023-10764-y

**Published:** 2023-03-28

**Authors:** Christine Deutschmann, Christian F. Singer, Daphne Gschwantler-Kaulich, Georg Pfeiler, Carmen Leser, Pascal A. T. Baltzer, Thomas H. Helbich, Christine Kraus, Ricarda Korbatits, Alaa Marzogi, Paola Clauser

**Affiliations:** 1 Department of Obstetrics and Gynecology, Division of General Gynecology and Gynecologic Oncology, Medical University of Vienna, Waehringer Guertel 18-20, 1090 Vienna, Austria; 2 Department of Biomedical Imaging and Image-Guided Therapy, Division of General and Pediatric Radiology, Medical University of Vienna, Waehringer Guertel 18-20, 1090 Vienna, Austria

**Keywords:** Residual fibroglandular breast tissue, Mastectomy, Local recurrence, New primary tumor

## Abstract

**Background:**

Residual fibroglandular breast tissue (RFGT) following a mastectomy has been claimed to be associated with the occurrence of an in-breast local recurrence (IBLR) or new primary tumor (NP). Yet, scientific evidence proving this assumption is lacking. The primary aim of the study was to verify whether RFGT following a mastectomy is a risk factor for an IBLR or NP.

**Methods:**

This retrospective analysis included all patients that underwent a mastectomy and were followed up at the Department of Obstetrics and Gynecology of the Medical University of Vienna between 01.01.2015 and 26.02.2020. RFGT volume (assessed on magnetic resonance imaging) was correlated with the prevalence of an IBLR and a NP.

**Results:**

A total of 105 patients (126 breasts) following a therapeutic mastectomy were included. After a mean follow-up of 46.0 months an IBLR had occurred in 17 breasts and a NP in 1 breast. A significant difference in RFGT volume was observed between the disease-free cohort and the subgroup with an IBLR or NP (*p* = .017). A RFGT volume of ≥ 1153 mm^3^ increased the risk by the factor 3.57 [95%CI 1.27; 10.03].

**Conclusions:**

RFGT volume is associated with an increased risk for an IBLR or NP.

**Supplementary Information:**

The online version contains supplementary material available at 10.1186/s12885-023-10764-y.

## Background

Local recurrence rates in women with breast cancer treated with a therapeutic simple (SME), nipple-sparing (NSM) or skin-sparing mastectomy (SSM) range from 7.9% [1] to 11.4% [2]

Even after prophylactic mastectomy, breast cancer incidence rates vary between 0 and 1.9% following bilateral risk-reducing mastectomy and 0 to 1.6% after contralateral risk-reducing mastectomy, respectively [3–8].

Any presumed remaining local oncological risk following the ablative operation has been attributed to residual fibroglandular tissue (RFGT) [1, 2, 9]. Concordantly, 60% to 80% of locoregional recurrences after mastectomy are located within the chest wall most commonly within the skin and subcutaneous tissue indicating an origin from RFGT, followed by recurrences within the pectoral muscle [3, 10].

The presence of RFGT following a mastectomy has been addressed by numerous studies [11–17]. Woitek et al. [11], for instance, detected RFGT in postoperative breast magnetic resonance imaging (MRI) scans following NSM and SSM in 20% of all breasts and significantly more frequently in patients with NSM than SSM. RFGT ranged from 0.5% to 26% of the preoperative fibroglandular tissue (FGT) with higher numbers after NSM than SSM.

The putative association of RFGT and local recurrence risk pressures surgeons to perform radical resection of breast parenchyma in the course of mastectomy in order to ensure complete breast tissue removal [11]. However, this poses the risk of too radical excisions with additional removal of subcutaneous fat tissue leading to ischemic mastectomy flaps.

Concordantly, Frey JD et al. [18] found the postoperative NSM flap thickness to be significantly less compared to preoperative measurements on MRI. Notably, a NSM flap thickness of less than 8.0 mm was identified as an independent predictor of ischemic complications.

Likewise, Roy De Vita et al. [19] found a statistically significant association between complications following NSMs and skin flaps of less than 5 mm.

Yet, it has to be noted that, the putative oncological risk attributed to RFGT has – to the best of our knowledge – only been suspected not statistically verified in literature.

Contrary to this assumption, Grinstein et al. observed 2 breast cancers in 169 BRCA1 and 2 mutation carriers following a mastectomy in areas without RFGT as visualized on breast MRI [17]. Similarly, Giannoti et al. [15] reported a local recurrence rate of 5.4% following therapeutic mastectomy and none in a risk-reducing mastectomy group. Yet, the therapeutic mastectomy subgroup had less RFGT compared to the prophylactic mastectomy cohort.

Statistical evaluation of the impact of RFGT following a mastectomy on the oncologic outcome of patients is of high scientific interest in order to possibly prevent surgical overtreatment in the future.

## Aims

The primary aim of the study was to assess whether RFGT volume following a therapeutic or prophylactic mastectomy is a risk factor for the occurrence of an in-breast local recurrence or a new-primary tumor.

Furthermore, the impact of the ratio of RFGT to the preoperative breast tissue volume as well as the impact of post-mastectomy radiotherapy with regard to RFGT volume on the oncologic outcome were assessed.

## Methods

### Inclusion and exclusion criteria

All patients following a therapeutic or prophylactic mastectomy that were presented in the tumor board of the Department of Obstetrics and Gynecology of the Medical University of Vienna, Austria, between 01.01.2015 and 26.02.2020 and had an archived postoperative breast magnetic resonance imaging (MRI) examination were included in the analysis.

Patients with a second-look resection owing to positive resection margins, a lipofilling procedure or flap reconstruction prior to the postoperative breast MRI as well as patients without available post-mastectomy MRI or clinical data were excluded from the study.

### Image acquisition and analysis

Breast MRI examinations were performed according to international guidelines [20] on 1.5 T or 3 T scanner, with dedicated coils and patients lying in a prone position. Measurements were performed by an experienced breast radiologist with more than 8 years of expertise in breast MRI.

Pre-contrast T1-weighted 3D gradient-echo sequences were used to measure RFGT. Fat tissue has a high signal intensity on non-fat saturated T1-weighted sequences, while fibroglandular tissue (FGT) has an intermediate to low signal intensity. On fat-saturated T1-weighted sequences, fat has a low signal intensity while FGT has an intermediate signal intensity, thus it is feasible to differentiate the two tissues with an acceptable accuracy. In the cases in which the differentiation between fat, FGT and scar after surgery was challenging on T1-weighted sequences, T2-weighted Turbo-Spin Echo sequences were used for reference to ensure a correct visual identification of RFGT. Furthermore, when available, preoperative MRI were used to aid in the correct identification of RFGT.

MRI measurements included whole breast, fibroglandular tissue (FGT) and RFGT volume. Volumes were calculated using semi-automated segmentation with a dedicated software (ITK-SNAP) [21] (see Figure S1). The system allows to segment separately different anatomical structures depending on their different signal intensities on imaging. Before starting with the segmentation, thresholds were defined to exclude anatomical areas not of interest from the segmentation. One or multiple regions of interest (ROI) were drawn in the region of interest (whole breast, FGT, RFGT). The software can automatically enlarge the area of interest within all the regions with the same signal intensity as in the initial ROI. Visual evaluation of the selected area was performed after the automated procedure and gradual adjustments were performed, when needed.

Measures are presented in mm^3^.

In addition, RFGT thickness was measured in the retroareolar region, as well as in the medial and lateral aspect of the breast. The RFGT thickness was measured at the level of the nipple for the retroareolar region. For the RFGT in the medial and lateral aspect of the breast, a visual assessment of the breast was performed, and the measurements were collected in the areas where the highest amount of RFGT was identified. All measurements were performed on T1-weighted sequences and evaluated on the axial plane (see Figure S2). Measures are presented in mm.

### Clinical data collection

Patient, disease and treatment characteristics were obtained by retrospective chart review.

The study was approved by the institutional review board. Informed consent was waived owing to the retrospective nature of the study.

### Definition “in-breast local recurrence”, “locoregional recurrence” and “new-primary tumor”

A local recurrence, referred to as “in-breast local recurrence” in the present study was defined as breast cancer recurrence of the same breast cancer subtype as the primary breast tumor and located in the ipsilateral breast, potentially including the skin, the nipple-areola-complex and/or the chest wall.

A locoregional recurrence was defined as breast cancer recurrence of a breast cancer subtype similar to the primary breast tumor situated in the locoregional lymph nodes of the breast such as in the axillary, supraclavicular, infraclavicular or mammary interna region with potential additional involvement of the skin, the nipple-areolar-complex or the chest wall.

A new-primary tumor was defined as breast cancer of a subtype different to the primary breast tumor located in the ipsilateral breast potentially including the skin, the nipple-areola-complex and/or the chest wall. Furthermore, all breast cancers that occurred in the breast, the skin, the nipple-areola-complex and/or the chest wall following a prophylactic mastectomy were referred to as new-primary tumors.

In the present study – solely local recurrences, locoregional recurrences with an involvement of either the skin, the nipple-areolar-complex or the chest wall, and new primary tumors were considered for analysis. Locoregional recurrences with cancer formations solely in the lymph nodes were excluded from analysis as the primary study objective was to investigate a potential correlation between remaining breast tissue following a mastectomy and the (re)occurrence of cancer.

### Statistics

Statistical analysis was performed with IBM SPSS Statistics Version 22. A *p*-value of ≤ 0.05 was considered significant. In case of multiple testing the Bonferroni correction was applied. Mean, standard deviation, minimum, maximum, median and interquartile range were used to describe metric variables. Frequencies and percentages were evaluated to describe nominal scaled parameters. In the course of hypothesis generating testing Pearson correlation coefficient and Spearman's rank correlation coefficient (in case of skewed distribution of data) were calculated. The correlation between two nominal scaled variables was tested using Chi-square test – and if required—Fisher's exact test. To test the difference between metric variables between 2 groups the students T-test and in case of skewed distribution of data Mann–Whitney-U-test were applied. In case of more than 2 groups the Kruskal–Wallis test was used. The clinically relevant RFGT threshold (the value with the maximum Youden index, defined as sensitivity plus specificity minus 1) was calculated using the ROC curve. In the course of statistical model validation, a multiple linear regression was performed. To interpret the clinical relevance of results effect size measures such as Cohen's d were determined.

## Results

### Demographic and clinical information

**Fig. 1:**
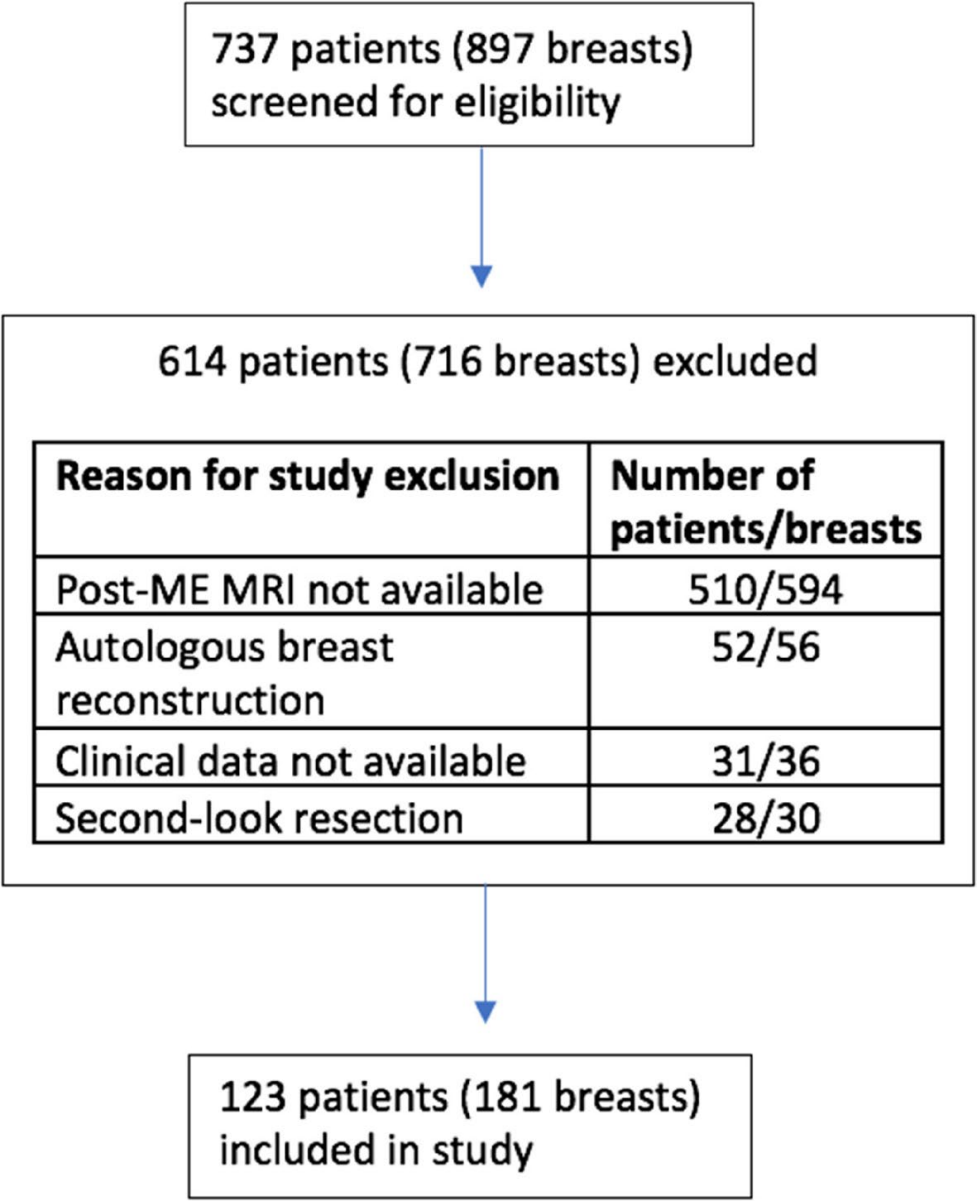
Study population

**Fig. 2:**
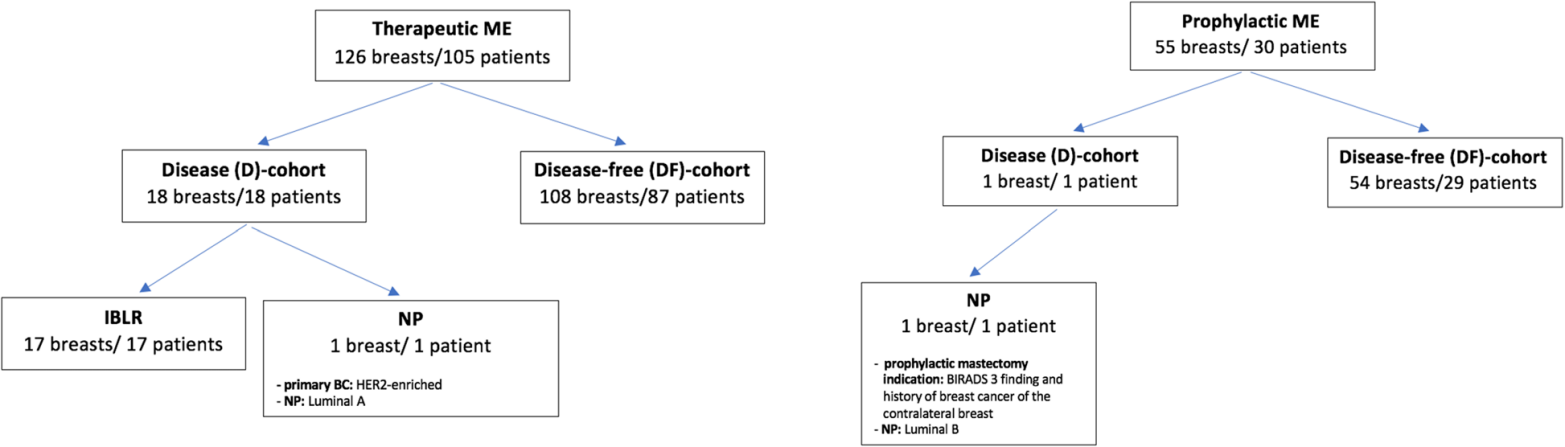
Oncologic outcome after therapeutic mastectomy (left figure) and after prophylactic mastectomy (right figure). ME Mastectomy, IBLR In-breast local recurrence, NP New primary

123 patients (181 breasts) were included in the study (see Fig. 1 and 2). In 126 breasts (105 patients) a therapeutic mastectomy was performed. Of these, most patients had a simple mastectomy (58.7%), followed by nipple-sparing (24.6%) and skin sparing mastectomy (16.7%). In the cohort with therapeutic mastectomy indication, an in-breast-local recurrence occurred in 17 breasts (17 patients) and a new primary in 1 breast (1 patient) (D-cohort). In 108 breasts (87 patients) no in-breast local recurrence or new primary occurred. The median follow-up time in the subgroup with a therapeutic mastectomy was 14.0 months (IQR 12.0; 19.2) in the DF-cohort and 25.3 months (12.8; 37.7) in the D-cohort.

In 55 breasts (30 patients) a prophylactic mastectomy was performed. Of these, the most frequent type of mastectomy was nipple-sparing mastectomy (63.6%), followed by skin-sparing mastectomy (23.6%) and simple mastectomy (12.7%). In the cohort with prophylactic mastectomy indication, a new primary occurred in 1 breast (1 patient) (D-cohort). In 54 breasts (29 patients) no new primary occurred (DF-cohort). The median follow-up time in the subgroup with a prophylactic mastectomy was 25.38 months (IQR 16.94; 41.26) in the DF-cohort and 74.81 months in the D-cohort.

Further demographic and clinical information is outlined in Table 1, 2 and 3.

**Table 1:** Demographics and disease characteristics I

	**THERAPEUTIC ME INDICATION**			**PROPHYLACTIC ME INDICATION**	
	**NO IBLR or NP** **(DF-cohort)** 87 patients/ 108 breasts	**WITH IBLR/NP (D-cohort)** 18 patients/ 18 breasts	***p*-VALUE**	**NO NP** **(DF-cohort)** 29 patients/ 54 breasts	**WITH NP** **(D-cohort)** 1 patient/ 1 breast
**Age** (mean years, SD)_PB_	50.3 ± 11.4	46.0 ± 17.1	.067	41.0 ± 9.4	66.2
**BMI** (mean kg/m^2^, SD)_PP_	25.56 ± 5.94	24.19 ± 3.58	.644	25.53 ± 5.26	NA
**Prior breast operation**_PB_	18 (16.7%)	6 (33.3%)	.110	9 (16.7%)	0
**Neoadjuvant chemotherapy (NACT)**_PP_	24 (27.6%)	4 (22.2%)	> .999		
**Radiotherapy(RT)**_PB_	26 (24.1%)	4 (22.2%)	> .999		
- Prior to ME	1 (0.9%)	1 (5.6%)	.266		
- Post ME	25 (23.1%)	3 (16.7%)	.761		
**Breast cancer subtype**_PB_			.931		
- Luminal A	31 (30.7%)	4 (25.0%)			
- Luminal B	20 (19.8%)	4 (25.0%)			
- HER2-enriched	33 (32.7%)	5 (31.2%)			
- Triple negative	17 (16.8%)	3 (18.8%)			

*PP* Analysis per patient, *PB* Analysis per breast, *IBLR* In-breast local recurrence, *NP* New primary tumor, *ME* Mastectomy, *NA* Not available, *SD* standard deviation

**Table 2:** Data regarding mastectomy and lymph node surgery

	**THERAPEUTIC ME INDICATION**			**PROPHYLACTIC ME INDICATION**	
	**NO IBLR or NP** **(DF-cohort)** 87 patients/ 108 breasts	**WITH IBLR/NP (D-cohort)** 18 patients/ 18 breasts	***p*-VALUE**	**NO NP** **(DF-cohort)** 29 patients/ 54 breasts	**WITH NP** **(D-cohort)** 1 patient/ 1 breast
**Mastectomy type**_PB_					
- Simple mastectomy (SME)	64 (59.3%)	10 (55.6%)	.201	6 (11.1%)	1 (100%)
- Nipple-sparing mastectomy (NSM)	24 (22.2%)	7 (38.9%)	.201	35 (64.8%)	0
- Skin-sparing mastectomy (SSM)	20 (18.5%)	1 (5.6%)	.201	13 (24.1%)	0
**Mastectomy indication**_PB_					
- **Oncologic**					
- Primary breast cancer	102 (94.4%)	15 (83.3%)	.119		
- Breast cancer recurrence	6 (5.6%)	3 (16.7%)	.119		
- **Prophylactic**^a^	16 (14.8%)	6 (33.3%)	.087		
- HBOC	16 (14.8%)	6 (33.3%)	.087	38 (70.4%)	0
- Mantle-field radiation	0	0		2 (3.7%)	0
- Undefined	0	0		14 (25.9%)	1 (100%)
**ME volume** (median cc, IQR)_PB_	455 (350;550)	305 (260;350)	.208	351 (181.5; 462.0)	NA
**Lymph node surgery**_PB_	95 (90.5%)	14 (87.5%)	.659		
- Sentinel node	45 (42.9%)	6 (37.5%)			
- Axillary dissection	50 (47.6%)	8 (50.0%)			

*PB* Analysis per breast, *IBLR* In-breast local recurrence, *NP* New primary tumor, *ME* Mastectomy, *NA* Not available, *IQR* Interquartile range

^a^ additionally to therapeutic indication

**Table 3:** Time between pre- and postoperative MRI, follow-up and time between mastectomy and occurrence of an IBLR or NP

	**THERAPEUTIC ME INDICATION**			**PROPHYLACTIC ME INDICATION**	
	**NO IBLR or NP** **(DF-cohort)** 87 patients/ 108 breasts	**WITH IBLR/NP (D-cohort)** 18 patients/ 18 breasts	***p*-VALUE**	**NO NP** **(DF-cohort)** 29 patients/ 54 breasts	**WITH NP** **(D-cohort)** 1 patient/ 1 breast
**Time between preoperative and postoperative MRI** (median months, IQR)_PB_	14.0 (12.0; 19.2)	25.3 (12.8; 37.7)	**.029**	14.47 (12.96; 22.77)	NA
**Follow-Up** (mean months, IQR)_PB_	36.9 (19.2; 56.8)	46.0 (22.8; 81.2)	.249	25.38 (16.94; 41.26)	74.81
**Time between mastectomy and occurrence of IBLR or NP** (median months, IQR)_PB_		35.1 (16.0; 57.6)			33.35

*PB* Analysis per breast, *IBLR* In-breast local recurrence, *NP* New primary tumor, *ME* Mastectomy, *NA* Not available, *IQR* Interquartile range

Details regarding cancer characteristics at diagnosis including grading, MIB, tumor stage, T- and N-stadium, DCIS, resection margin, lymphovascular invasion and extent of disease are shown as supplement material (see Table S1).

Details regarding breast reconstruction including time of reconstruction, type of prothesis and use of ADMs and synthetic meshes are available as supplement material (see Table S2).

### Breast MRI measurements

Details regarding MRI measurements are outlined in Table 4.

**Table 4:** MRI measurements including whole breast volume, fibroglandular tissue (FGT) and residual fibroglandular breast tissue (RFGT)

	**THERAPEUTIC ME INDICATION**			**PROPHYLACTIC ME INDICATION**	
	**NO IBLR/NP** **(DF cohort)**	**WITH IBLR/NP** **(D-cohort)**	***p*-VALUE**	**NO IBLR/NP (DF-cohort)**	**WITH IBLR/NP** **(D-cohort)**
**Whole breast volume** (median cm^3^, IQR) ^a^	615.80 (409.90; 851.10)	455.00 (250.20; 814.00)	.169	388.40 (217.10; 632.850)	NA
**Fibroglandular tissue (FGT)**					
- Volume (median cm^3^, IQR)	69.950 (33.68; 128.10)	40.255 (196.10; 139.70)	.351	50.31 (34.485; 78.565)	NA
**Residual fibroglandular tissue (RFGT)**					
- Medial (median mm, IQR)	0 (0; 1.0)	2.0 (1.0; 2.0)	**.003**	0 (0; 2.0)	NA
- Lateral (median mm, IQR)	1.0 (0; 3.0)	1.0 (1.0; 2.0)	.910	2.0 (1.0; 3.0)	NA
- Retroareolar (median mm, IQR)	1.0 (0; 2.0)	4.0 (3.0; 5.0)	**.029**	2.0 (0; 4.0)	NA
- Volume (median mm^3^, IQR)	155.0 (20.0; 1779.5)	1776.5 (107.0; 3152.0)	**.017**	693.50 (50.0; 2970.0)	100.0

All variables were analysed per breast. *IBLR* In-breast local recurrence, *NP* New primary tumor

^a^including skin, subcutaneous fat and fibroglandular breast tissue measured preoperatively

RFGT was detected in 88.9% of breasts following a therapeutic ME and 92.7% of breasts following a prophylactic ME.

Including all patients following a therapeutic mastectomy, a statistically higher RFGT thickness was seen in the D-cohort compared to the DF-cohort regarding the medial (2.0 mm (IQR 1.0–2.0) vs. 0 mm (IQR 0; 1.0); *p* = 0.003; r = 0.36 (moderate effect)) and retroareolar measurements (4.0 mm (3.0; 5.0) vs. 1.0 mm (0; 2.0); *p* = 0.029, r = 0.27 (weak effect)). No statistically significant difference in RFGT thickness was seen between the two cohorts considering the lateral measurements (1.0 mm (IQR 1.0–2.0) vs. 1.0 mm (IQR 0–3.0); *p* = 0.910).

Including all patients following a prophylactic mastectomy, no comparisons of RFGT thickness and RFGT volume were performed between the D- and DF-cohort owing to a small sample size and missing data.

### RFGT volume

Comprising all patients with a therapeutic mastectomy, a significantly higher RFGT volume was seen in the D- compared to the DF-cohort (1776.5 mm^3^ (107.0; 3152.0) vs. 155.0 mm^3^ (IQR 20.0; 1779.5); *p* = 0.017; r = 0.21 (weak effect)) in univariate analysis.

RFGT volume represented 4.41% of the preoperative fibroglandular tissue (FGT) volume in the D-cohort and 0.22% in the DF-cohort.

Using the ROC curve we calculated the Youden index in order to define the threshold for a clinically relevant RFGT volume of 1153 mm^3^ (YI = 0.352; sensitivity = 0.611; specificity = 0.694). 34.1% of all cases following a therapeutic mastectomy had a RFGT volume above this threshold resulting in an increased risk for an IBLR or NP by the factor 3.57 (OR = 3.571; [95% CI 1.27; 10.03]; *p* = 0.012).

### The impact of the ratio of RFGT volume to FGT volume on the oncologic outcome

We furthermore looked at the impact of the ratio of RFGT volume to FGT volume on the occurrence of an IBLR or NP. Considering all breasts following a therapeutic mastectomy, the ratio of RFGT volume to FGT volume was marginally significantly associated with the occurrence of an IBLR or NP. Higher numbers of IBLR or NP were observed in breasts with a higher RFGT volume/FGT volume-ratio (1.41% vs. 0.38%; *p* = 0.084).

We calculated the Youden Index using the ROC curve in order to define the threshold for a clinically relevant RFGT volume/FGT volume ratio of 0.7% (YI = 0.344; *p* = 0.084; [95% CI 51.5%; 77.4%]; sensitivity = 0.224; specificity = 0.941). 49% of breasts following a therapeutic mastectomy had a RFGT volume/FGT volume ratio above this threshold leading to an increased risk for an IBLR or NP by the factor 4.63 (OR = 4.63; [95% CI 1.21; 17.79]; *p* = 0.017).

### The impact of post-mastectomy radiotherapy (PMRT) on the oncologic outcome with regard to RFGT volume

We furthermore assessed the impact of PMRT on the occurrence of an IBLR or NP with regard to the RFGT volume. When allocating all breasts following a TME to one of two cohorts based on RFGT volume (breasts with comparatively high RFGT volume (> median 266.50 mm^3^) versus breasts with lower RFGT volume (≤ median 266.50 mm^3^)) a difference in the IBLR or NP rate between breasts following PMRT compared to no PMRT was particularly evident in the cohort with high RFGT volumes (high RFGT volume: 17% vs. 22%; low RFGT volume: 6% vs. 9%), yet not statistically significant.

### Other factors associated with the oncologic outcome

In contrast to RFGT volume, none of the following parameters showed a significant association with the occurrence of an IBLR or NP in univariate analysis: age at diagnosis (*p* = 0.067), HBOC (*p* = 0.087), intrinsic subtype (*p* = 0.931), invasive lobular subtype (*p* = 0.761), tumor stage (*p* = 0.313), T-stadium (*p* = 0.611), N-stadium (*p* = 0.944), grading (*p* = 0.200), DCIS (*p* > 0.999), lymphovascular invasion (*p* = 0.704), positive/negative resection margin (*p* = 0.621), closest resection margin (*p* = 0.419), MIB (*p* = 0.936), disease extent (*p* = 0.238) and post-mastectomy radiotherapy (*p* = 0.761).

## Discussion

The present study aimed to assess whether residual fibroglandular breast tissue following a therapeutic or prophylactic mastectomy is a risk factor for the occurrence of an in-breast local recurrence (IBLR) or new-primary tumor (NP).

Including all patients following a therapeutic mastectomy, significantly higher RFGT volumes were observed in the D-cohort (disease-cohort including patients with an IBLR or NP) compared to the DF-cohort (disease-free cohort including patients without an IBLR or NP) (*p* = 0.017). Concordantly, a statistically higher RFGT thickness was seen in the D-cohort compared to the DF-cohort (medial measurements: *p* = 0.003; retroareolar measurements: *p* = 0.029).

Consequently, residual fibroglandular breast tissue is associated with a remaining local oncologic risk following a mastectomy. We defined a threshold of 1153 mm^3^ as clinically relevant RFGT volume (YI = 0.352; sensitivity = 0.611; specificity = 0.694) resulting in an increased risk for an IBLR or NP by the factor 3.57 (OR = 3.571; [95% CI 1.27; 10.03]; *p* = 0.012).

Known risk factors for a local or locoregional recurrence include younger age, premenopausal status, disease stage, T and N-stage, high grade, Ki67 overexpression, ER negative and PR negative expression status, triple negative and HER2-enriched subtype, lymphovascular invasion, microinvasion, multifocality and multicentricity, positive resection margin and margin proximity [22–27].

This is – to the best of our knowledge – the first study to prove an association of RFGT with the risk of a local recurrence or new primary tumor. Notably, other parameters well known to be associated with increased rates of tumor recurrence were not found to be so in this study. We ascribe this to the small sample size and the short follow-up duration.

RFGT was observed in 88.9% of breasts following a therapeutic mastectomy and 92.7% of breasts following a prophylactic mastectomy, respectively. Similar to the present study detection rates of RFGT have been described between 5 and 100% in literature [28].

The median percentage of RFGT compared to preoperative FGT volume was 4.41% in the D-cohort and 0.22% in the DF cohort. In comparison Woitek, et al. [11] found a mean percentage of unremoved FGT between 5.8 ± 8.2% of the preoperative breast tissue volume.

We studied the impact of the ratio of RFGT volume to FGT volume on the occurrence of an IBLR or NP and found higher numbers of IBLR or NP in breasts with a higher RFGT volume/FGT volume-ratio (1.41% vs. 0.38%; *p* = 0.084). We defined a threshold of 0.7% as a clinically relevant RFGT volume/FGT volume ratio leading to an elevation of the risk for an IBLR or NP by the factor 4.63 (OR = 4.63; [95% CI 1.21; 17.79]; *p* = 0.017).

Lastly, we assessed the impact of radiotherapy following a therapeutic mastectomy on the occurrence of an IBLR or NP with regard to RFGT volume. Including all breasts following a therapeutic mastectomy the proportion of breasts with an IBLR or NP was numerically higher if no PMRT was conducted. This difference was even more evident in breasts with high RFGT volumes (> median 266.50 mm^3^).

Yet, owing to the small sample size solely a numerical trend and no statistical significance of the benefit of PMRT on the oncologic outcome of patients following a therapeutic mastectomy with remaining fibroglandular breast tissue could be shown. As radiotherapy is known to eliminate tumor foci remaining in the locoregional tissue [29] the suggested positive impact of PMRT in this analysis on the disease-free survival of patients in the presence of high RFGT volumes following a therapeutic mastectomy is worth further studying.

Moreover, as previously suggested, the presence and location of RFGT should be documented in postoperative breast imaging reports in order to allow individual planning of PMRT according to these “high-risk” volumes and guide patient surveillance [28].

Following a therapeutic mastectomy an IBLR occurred in 13.5% of breasts (17/126 breasts, 17/105 patients) and a NP in 0.8% of breasts (1/126 breasts, 1/105 patients). The median follow-up-period in this cohort was 46.0 months (IQR 22.8; 81.2). The median time between mastectomy and occurrence of an IBLR or NP was 35.1 months (IQR 16.0; 57.6).

Following a prophylactic mastectomy, a NP occurred in 1.8% of breasts (1/55 breasts, 1/30 patients). The follow-up time in this patient was 74.81 months. The time between prophylactic mastectomy and occurrence of the NP was 33.35 months.

In comparison, local recurrence rates following SME, NSM and SSM are reported to range between 7.9% and 11.4% in literature [1, 2, 9]. The comparatively high rates of disease recurrence in the present study can be attributed to the inclusion criterion of an archived breast MRI for RFGT determination MRI is the imaging method with highest measuring accuracy [28]. Yet, as MRI is only performed for specific indications in aftercare following a mastectomy such as the presumptive diagnosis of a cancer reoccurrence, a selection bias of the included patient population and the reporting of a surpassingly high rate of IBLRs and NPs, respectively, could be possible.

The following additional limitations of the study have to be discussed:

In the absence of a validated software or method to measure FGT and RFGT on imaging, the authors in consensus determined the methodology. A single reader performed all the measurement using the same methodology, to ensure comparability of the measurements between the included subjects.

It has to be noted, that in case of the presence of an IBLR or NP in the post-mastectomy MRI scan RFGT was measured sparing the tumor tissue. Yet, as the IBLR or NP most likely contained breast tissue— an underestimation of RFGT volume in the D-cohort and underreporting of the difference of RFGT volume between the DF- and D-cohort could be imaginable.

Owing to the low incidence of a NP following a therapeutic mastectomy separate statistical analyses of the impact of RFGT on the occurrence of an IBLR versus a NP could not be conducted.

Due to the small sample size further statistical comparisons of RFGT measurements between the DF- and D-cohort in the patient population following a prophylactic mastectomy could not be performed.

## Conclusions

In conclusion, comprising all breasts following a therapeutic mastectomy, RFGT volume was significantly higher in the cohort of patients with an IBLR or NP compared to the disease-free subgroup. Consequently, residual fibroglandular breast tissue is associated with a remaining local oncologic risk following a mastectomy.

Surgeons should be aware of risk factors that increase the likelihood for RFGT when performing a mastectomy such as: older age, younger age (potentially owing to the higher rate of NSM and risk-reducing mastectomies in this group), higher body mass index, parity (versus nulliparity), larger preoperative fibroglandular breast tissue volume, indication for mastectomy (risk-reducing > therapeutic), type of mastectomy (NSM > SSM > simple mastectomy), larger skin envelope thickness and lower surgical experience [11, 12, 15, 17, 30].

## Supplementary Information

**Additional file 1: Figure S1.** FGT volume. Example of a segmentation performed in a right breast before mastectomy with the software ITK-SNAP. A threshold was defined to select the intensity of fibroglandular tissue (FGT) and multiple regions of interest were position in order to segment the whole area of FGT. The segmentation of the volume was performed automatically and the final volume was revised by the reader (board certified breast radiologist). The final measured volume is shown in red.

**Additional file 2: Figure S2.** RFGT. Example of a measurement of residual fibroglandular tissue (RFGT) after nipple sparing mastectomy and reconstruction with an implant. RFGT was identified and measured in the retroareolar region and in the lateral part of the breast.

**Additional file 3: Table S1.** Demographics and disease characteristics II, all variables were analysed per breast, *IBLR* In-breast local recurrence, *NP* New primary tumor, *DF-Cohort* Disease free cohort, *D-cohort* Disease cohort, *IQR* interquartile range.

**Additional file 4: Table S2.** Reconstruction data, all variables were analysed per breast, *IBLR* In-breast local recurrence, *NP* New primary tumor, *DF-cohort* Disease free cohort, *D-cohort* Disease cohort.

## Data Availability

The datasets generated and analysed during the current study are not publicly available due to ongoing data analysis and further manuscript preparation on additional aspects of the topic but are available from the corresponding author on reasonable request.

## Abbreviations

D-cohort: Disease cohort
DF-cohort: Disease-free cohort
FGT: Fibroglandular tissue
IBLR: In-breast local recurrence
NP: New primary tumor
NSM: Nipple-sparing mastectomy
PMRT: Post-mastectomy radiotherapy
RFGT: Residual fibroglandular breast tissue
SME: Simple mastectomy
SSM: Skin-sparing mastectomy
TME: Therapeutic mastectomy

## References

[1] Al-Himdani S (2016). Prediction of margin involvement and local recurrence after skin-sparing and simple mastectomy. Eur J Surg Oncol.

[2] De La Cruz L (2015). Overall survival, disease-free survival, local recurrence, and nipple-areolar recurrence in the setting of nipple-sparing mastectomy: a meta-analysis and systematic review. Ann Surg Oncol.

[3] Kaas R (2010). Prophylactic mastectomy in BRCA1 and BRCA2 mutation carriers: very low risk for subsequent breast cancer. Ann Surg.

[4] Mutter RW (2015). Breast cancer after prophylactic mastectomy (bilateral or contralateral prophylactic mastectomy), a clinical entity: presentation, management, and outcomes. Breast Cancer Res Treat.

[5] Rebbeck TR (2004). Bilateral prophylactic mastectomy reduces breast cancer risk in BRCA1 and BRCA2 mutation carriers: the PROSE study group. J Clin Oncol.

[6] Domchek SM (2010). Association of risk-reducing surgery in BRCA1 or BRCA2 mutation carriers with cancer risk and mortality. JAMA.

[7] Heemskerk-Gerritsen BA (2013). Substantial breast cancer risk reduction and potential survival benefit after bilateral mastectomy when compared with surveillance in healthy BRCA1 and BRCA2 mutation carriers: a prospective analysis. Ann Oncol.

[8] Skytte AB (2011). Breast cancer after bilateral risk-reducing mastectomy. Clin Genet.

[9] Zhou X, Li Y (2016). Local recurrence after breast-conserving surgery and mastectomy following neoadjuvant chemotherapy for locally advanced breast cancer - a meta-analysis. Breast Care (Basel).

[10] Heemskerk-Gerritsen BA (2007). Prophylactic mastectomy in BRCA1/2 mutation carriers and women at risk of hereditary breast cancer: long-term experiences at the Rotterdam Family Cancer Clinic. Ann Surg Oncol.

[11] Woitek R (2018). MRI-based quantification of residual fibroglandular tissue of the breast after conservative mastectomies. Eur J Radiol.

[12] Torresan RZ (2005). Evaluation of residual glandular tissue after skin-sparing mastectomies. Ann Surg Oncol.

[13] Dreadin J (2012). Risk of residual breast tissue after skin-sparing mastectomy. Breast J.

[14] Zippel D (2015). Magnetic resonance imaging (MRI) evaluation of residual breast tissue following mastectomy and reconstruction with silicone implants. Clin Imaging.

[15] Giannotti DG (2018). Analysis of skin flap thickness and residual breast tissue after mastectomy. Int J Radiat Oncol Biol Phys.

[16] Tewari M (2004). Residual breast tissue in the skin flaps after Patey mastectomy. Indian J Med Res.

[17] Grinstein O (2019). Residual Glandular Tissue (RGT) in BRCA1/2 germline mutation carriers with unilateral and bilateral prophylactic mastectomies. Surg Oncol.

[18] Frey JD (2017). Mastectomy flap thickness and complications in nipple-sparing mastectomy: objective evaluation using magnetic resonance imaging. Plast Reconstr Surg Glob Open.

[19] De Vita R (2017). Outcome evaluation after 2023 nipple-sparing mastectomies: our experience. Plast Reconstr Surg.

[20] Sardanelli F (2010). Magnetic resonance imaging of the breast: recommendations from the EUSOMA working group. Eur J Cancer.

[21] Yushkevich PA (2006). User-guided 3D active contour segmentation of anatomical structures: significantly improved efficiency and reliability. Neuroimage.

[22] Li Q (2014). Risk factors for locoregional recurrence after postmastectomy radiotherapy in breast cancer patients with four or more positive axillary lymph nodes. Curr Oncol.

[23] Karlsson P (2012). Patterns and risk factors for locoregional failures after mastectomy for breast cancer: an international breast cancer study group report. Ann Oncol.

[24] Petit JY (2012). Risk factors associated with recurrence after nipple-sparing mastectomy for invasive and intraepithelial neoplasia. Ann Oncol.

[25] Mamtani A (2019). Impact of age on locoregional and distant recurrence after mastectomy for ductal carcinoma in situ with or without microinvasion. Ann Surg Oncol.

[26] Glorioso JM (2017). Margin proximity correlates with local recurrence after mastectomy for patients not receiving adjuvant radiotherapy. Ann Surg Oncol.

[27] Fitzsullivan E (2013). Incidence and consequence of close margins in patients with ductal carcinoma-in situ treated with mastectomy: is further therapy warranted?. Ann Surg Oncol.

[28] Kaidar-Person O (2020). Residual glandular breast tissue after mastectomy: a systematic review. Ann Surg Oncol.

[29] Ebctcg (2014). Effect of radiotherapy after mastectomy and axillary surgery on 10-year recurrence and 20-year breast cancer mortality: meta-analysis of individual patient data for 8135 women in 22 randomised trials. Lancet.

[30] Papassotiropoulos B (2019). Prospective evaluation of residual breast tissue after skin- or nipple-sparing mastectomy: results of the SKINI-Trial. Ann Surg Oncol.

