# Supplementary material for: Residual fibroglandular breast tissue after mastectomy is associated with an increased risk of a local recurrence or a new primary breast cancer"
Source: BMC Cancer. 2023 Mar 28;23:281. doi: 10.1186/s12885-023-10764-y (PMC10044359; doi:10.1186/s12885-023-10764-y)
Supplement: Supplementary file 1 — Additional file 1: Figure S1. FGT volume. Example of a segmentation performed in a right breast before mastectomy with the software ITK-SNAP. A threshold was defined to select the intensity of fibroglandular tissue (FGT) and multiple regions of interest were position in order to segment the whole area of FGT. The segmentation of the volume was performed automatically and the final volume was revised by the reader (board certified breast radiologist). The final measured volume is shown in red. [file 12885_2023_10764_MOESM1_ESM.docx]

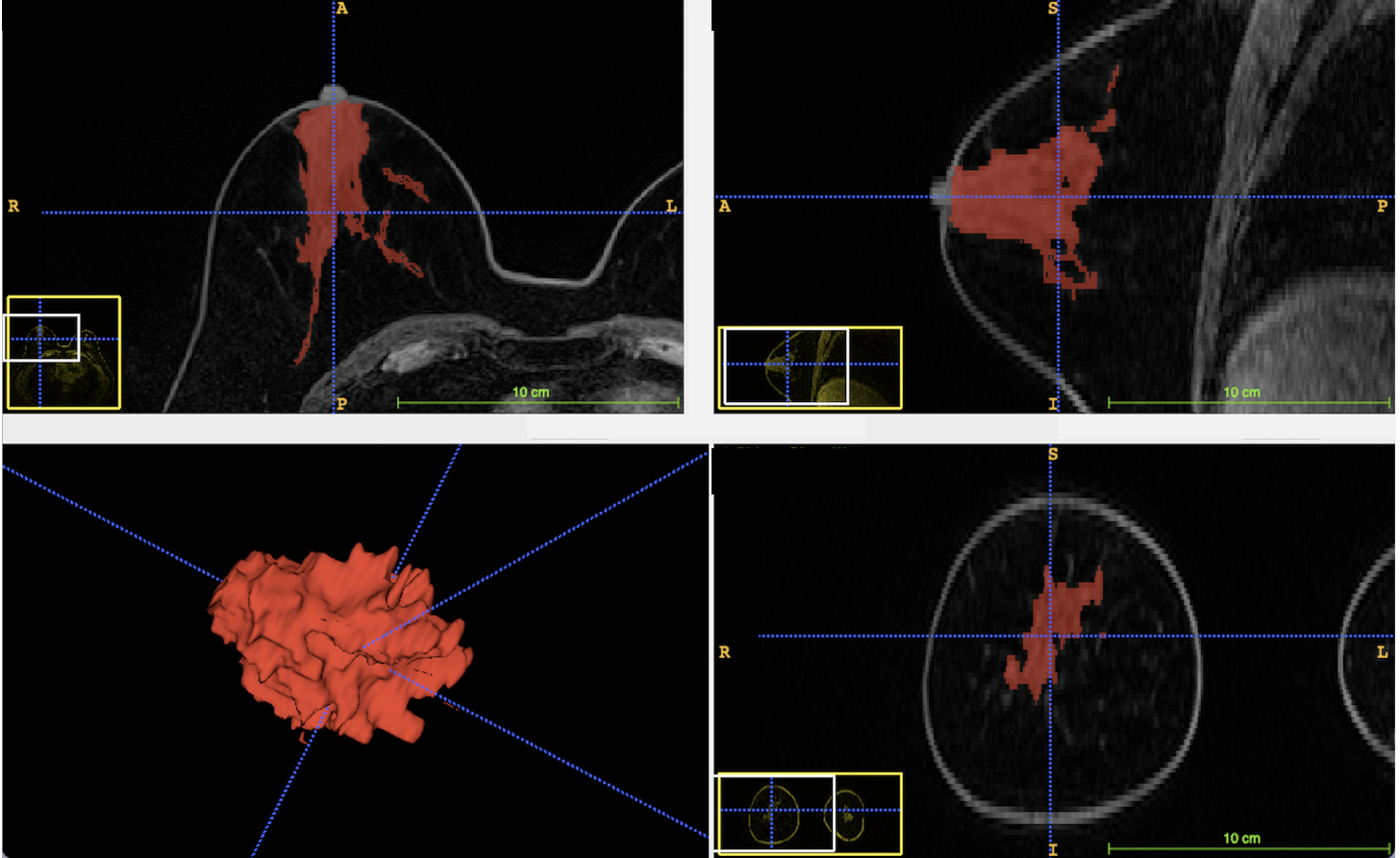


Figure S1: FGT volume. Example of a segmentation performed in a right breast before mastectomy with the software ITK-SNAP. A threshold was defined to select the intensity of fibroglandular tissue (FGT) and multiple regions of interest were position in order to segment the whole area of FGT. The segmentation of the volume was performed automatically and the final volume was revised by the reader (board certified breast radiologist). The final measured volume is shown in red.
