# Supplementary material for: Residual fibroglandular breast tissue after mastectomy is associated with an increased risk of a local recurrence or a new primary breast cancer"
Source: BMC Cancer. 2023 Mar 28;23:281. doi: 10.1186/s12885-023-10764-y (PMC10044359; doi:10.1186/s12885-023-10764-y)
Supplement: Supplementary file 2 — Additional file 2: Figure S2. RFGT. Example of a measurement of residual fibroglandular tissue (RFGT) after nipple sparing mastectomy and reconstruction with an implant. RFGT was identified and measured in the retroareolar region and in the lateral part of the breast. [file 12885_2023_10764_MOESM2_ESM.docx]

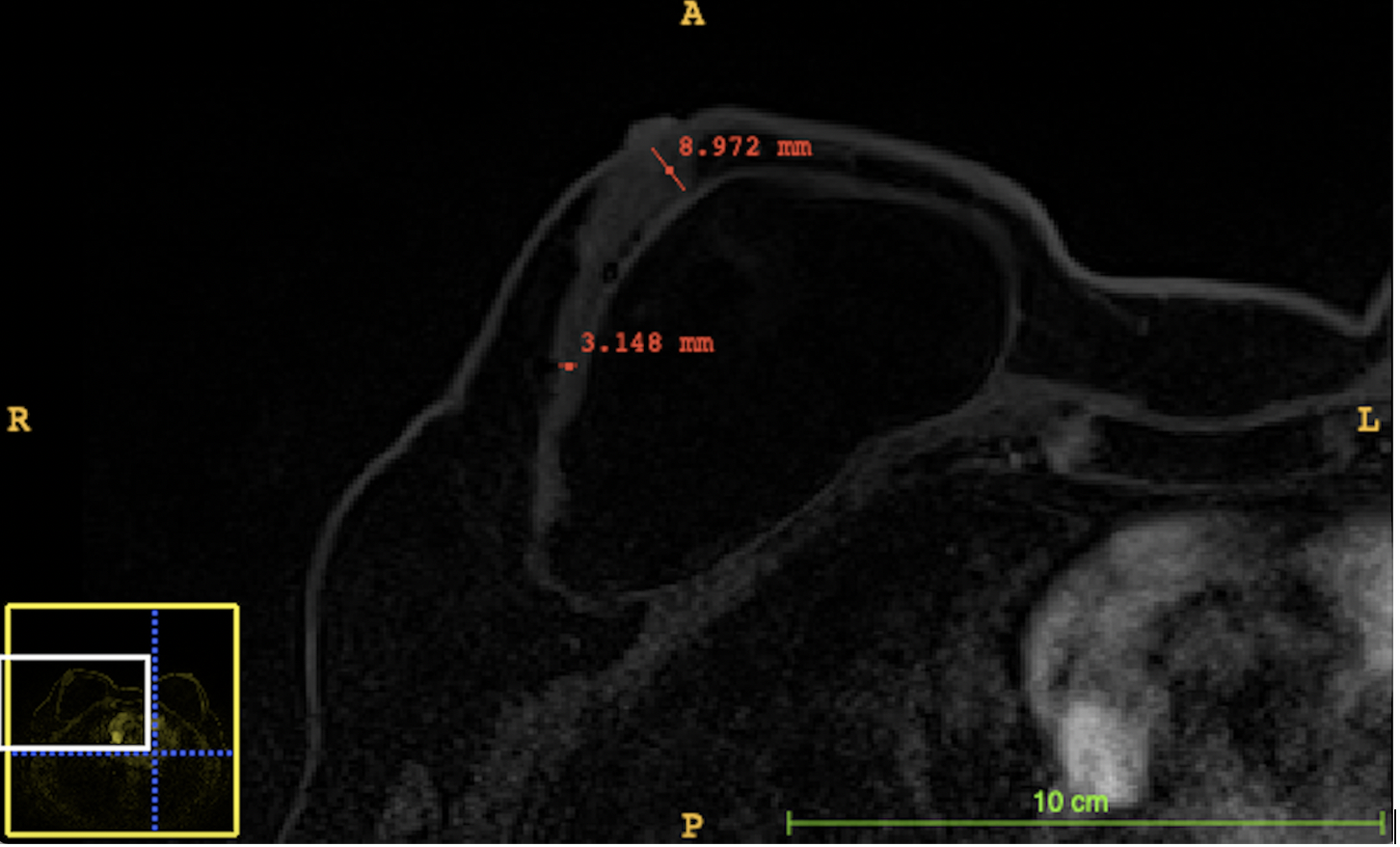


Figure S2: RFGT. Example of a measurement of residual fibroglandular tissue (RFGT) after nipple sparing mastectomy and reconstruction with an implant. RFGT was identified and measured in the retroareolar region and in the lateral part of the breast.
