# Supplementary material for: Residual fibroglandular breast tissue after mastectomy is associated with an increased risk of a local recurrence or a new primary breast cancer"
Source: BMC Cancer. 2023 Mar 28;23:281. doi: 10.1186/s12885-023-10764-y (PMC10044359; doi:10.1186/s12885-023-10764-y)
Supplement: Supplementary file 3 — Additional file 3: Table S1. Demographics and disease characteristics II, all variables were analysed per breast, IBLR In-breast local recurrence, NP New primary tumor, DF-Cohort Disease free cohort, D-cohort Disease cohort, IQR interquartile range. [file 12885_2023_10764_MOESM3_ESM.docx]

| **TABLE S2: RECONSTRUCTION DATA** | **THERAPEUTIC ME INDICATION** | | | **PROPHYLACTIC ME INDICATION** | |
| --- | --- | --- | --- | --- | --- |
|  | **NO IBLR/NP**  **(DF-cohort)**  87 patients/  108 breasts | **WITH IBLR/NP**  **(D-cohort)**  18 patients/  18 breasts | **p-VALUE** | **NO IBLR/NP**  **(DF-cohort)**  29 patients/  54 breasts | **WITH IBLR/NP**  **(D-cohort)**  1 patient/  1 breast |
| **Time of reconstruction and**  **type of prothesis** |  |  | > .999 |  |  |
| - Immediate, primary-fixed  volume implant | 40 | 6 |  | 44 | 0 |
| - Immediate, primary tissue  expander | 13 | 2 |  | 5 | 0 |
| - Delayed, primary tissue  expander | 5 | 0 |  | 0 | 0 |
| - No reconstruction | 50 | 10 |  | 5 | 1 |
| **ADM and synthetic mesh** |  |  | .045 |  |  |
| - ADM | 18 | 6 |  | 23 | 0 |
| - Synthetic mesh | 19 | 0 |  | 19 | 0 |
| - None  (Numbers shown for breasts with  reconstruction) | 17 | 2 |  | 5 | 0 |

Table S2: Reconstruction data, all variables were analysed per breast, IBLR…in-breast local recurrence, NP…new primary tumor, DF-cohort…disease free cohort, D-cohort…disease cohort
