# Supplementary material for: Residual fibroglandular breast tissue after mastectomy is associated with an increased risk of a local recurrence or a new primary breast cancer"
Source: BMC Cancer. 2023 Mar 28;23:281. doi: 10.1186/s12885-023-10764-y (PMC10044359; doi:10.1186/s12885-023-10764-y)
Supplement: Supplementary file 4 — Additional file 4: Table S2. Reconstruction data, all variables were analysed per breast, IBLR In-breast local recurrence, NP New primary tumor, DF-cohort Disease free cohort, D-cohort Disease cohort. [file 12885_2023_10764_MOESM4_ESM.docx]

| **TABLE S1: DEMOGRAPHICS AND**  **DISEASE CHARACTERISTICS II** | **THERAPEUTIC ME INDICATION** | | |
| --- | --- | --- | --- |
|  | **NO IBLR/NP**  **(DF-cohort)**  87 patients/  108 breasts | **WITH IBLR/NP**  **(D-cohort)**  18 patients/  18 breasts | **p-VALUE** |
| **Breast cancer characteristics at diagnosis** |  |  |  |
| - **DCIS** | 39 (37.1%) | 6 (33.3%) | .757 |
| - DCIS without microinvasion | 35 (33,3%) | 6 (33,3%) |  |
| - DCIS with microinvasion | 4 (3.8%) | 0 |  |
| - none | 66 (62,9%) | 12 (66,7%) |  |
| - **Grading** |  |  | .390 |
| - G1 | 13 (12,6%) | 1 (5,9%) |  |
| - G2 | 47 (45,6%) | 6 (35,3%) |  |
| - G3 | 43 (41,7%) | 10 (58,8%) |  |
| - **MIB** |  |  | .989 |
| ≤ 20% | 44 (43,6%) | 7 (43,8%) |  |
| > 20% | 57 (56,4%) | 9 (56,2%) |  |
| - **Stage** |  |  | .409 |
| - Tis N0 M0 | 20 (20,8%) | 0 |  |
| - T1 N0 M0 | 26 (27,1%) | 5 (38,5%) |  |
| - T0/T1, N1, M0 | 17 (17,7%) | 4 (30,8%) |  |
| - T2, N1, M0  - T3, N0, M0 | 5 (5,2%) | 0 |  |
| - T0/T1, N2, M0  - T2, N2, M0  - T3, N1/N2, M0 | 7 (7,3%) | 0 |  |
| - T4, N0/N1/N2, M0 | 1 (1%) | 0 |  |
| - all T, N3, M0 | 2 (2,1%) | 0 |  |
| - all T, all N, M1 | 18 (18,8%) | 4 (30,8%) |  |
| - **T-Stage** |  |  | .480 |
| - T0 | 4 (4,0%) | 0 |  |
| - Tis | 20 (19,8%) | 1 (7,7%) |  |
| - T1mic | 3 (3,0%) | 0 |  |
| - T1a | 4 (4,0%) | 2 (15,4%) |  |
| - T1b | 6 (5,9%) | 2 (15,4%) |  |
| - T1c | 27 (26,7%) | 4 (30,8%) |  |
| - T2 | 27 (26,7%) | 4 (30,8%) |  |
| - T3 | 9 (8,9%) | 0 |  |
| - T4b | 1 (1,0%) | 0 |  |
| - **N-Stage** |  |  | .683 |
| - pN0 | 61 (57,5%) | 9 (56,3%) |  |
| - pN1 |  |  |  |
| - pN1mi | 4 (3,8%) | 1 (6,3%) |  |
| - pN1a | 17 (16,0%) | 3 (18,8%) |  |
| - pN2a | 10 (9,4%) | 0 |  |
| - pN3a | 3 (2,8%) | 1 (6,3%) |  |
| - pNx | 9 (8,5%) | 2 (12,5%) |  |
| - **R** |  |  | .621 |
| - R0 | 100 (95,2%) | 17 (94,4%) |  |
| - R1 | 4 (3,8%) | 1 (5,6%) |  |
| - **Closest resection margin** (median, IQR) | 5.0 (2; 10) | 5.0 (1.5; 8) | .419 |
| - **Lymphovascular invasion** |  |  | .704 |
| - L0 | 41 (62.1%) | 4 (50.0%) |  |
| - L1 | 25 (37.9%) | 4 (50.0%) |  |
| - **Extent of disease** |  |  | .238 |
| - bifocal | 7 (6.7%) | 0 |  |
| - multifocal | 21 (20.0%) | 1 (5.6%) |  |
| - multicentric | 28 (26.7%) | 8 (44.4%) |  |
| - unknown | 49 (46.7%) | 9 (50.0%) |  |

Table S1: Demographics and disease characteristics II, all variables were analysed per breast, IBLR…in-breast local recurrence, NP…new primary tumor, DF-cohort…disease free cohort, D-cohort…disease cohort, IQR…interquartile range
